# Perinatal Outcomes and Unconventional Natural Gas Operations in Southwest Pennsylvania

**DOI:** 10.1371/journal.pone.0126425

**Published:** 2015-06-03

**Authors:** Shaina L. Stacy, LuAnn L. Brink, Jacob C. Larkin, Yoel Sadovsky, Bernard D. Goldstein, Bruce R. Pitt, Evelyn O. Talbott

**Affiliations:** 1 Department of Environmental and Occupational Health, Graduate School of Public Health, University of Pittsburgh, Pittsburgh, Pennsylvania, United States of America; 2 Department of Epidemiology, Graduate School of Public Health, University of Pittsburgh, Pittsburgh, Pennsylvania, United States of America; 3 Magee-Womens Research Institute and Department of Obstetrics, Gynecology and Reproductive Sciences, School of Medicine, University of Pittsburgh, Pittsburgh, Pennsylvania, United States of America; Stony Brook University, Graduate Program in Public Health, UNITED STATES

## Abstract

Unconventional gas drilling (UGD) has enabled extraordinarily rapid growth in the extraction of natural gas. Despite frequently expressed public concern, human health studies have not kept pace. We investigated the association of proximity to UGD in the Marcellus Shale formation and perinatal outcomes in a retrospective cohort study of 15,451 live births in Southwest Pennsylvania from 2007–2010. Mothers were categorized into exposure quartiles based on inverse distance weighted (IDW) well count; least exposed mothers (first quartile) had an IDW well count less than 0.87 wells per mile, while the most exposed (fourth quartile) had 6.00 wells or greater per mile. Multivariate linear (birth weight) or logistical (small for gestational age (SGA) and prematurity) regression analyses, accounting for differences in maternal and child risk factors, were performed. There was no significant association of proximity and density of UGD with prematurity. Comparison of the most to least exposed, however, revealed lower birth weight (3323 ± 558 vs 3344 ± 544 g) and a higher incidence of SGA (6.5 vs 4.8%, respectively; odds ratio: 1.34; 95% confidence interval: 1.10–1.63). While the clinical significance of the differences in birth weight among the exposure groups is unclear, the present findings further emphasize the need for larger studies, in regio-specific fashion, with more precise characterization of exposure over an extended period of time to evaluate the potential public health significance of UGD.

## Introduction

Unconventional gas development (UGD), characterized by advances in engineering, including horizontal drilling and high volume hydraulic fracturing, enables extraction of large amounts of fossil fuel from shale deposits at depths that were previously unapproachable [1]. In Pennsylvania, UGD in the Marcellus Shale formation has rapidly advanced from only 44 such wells known to be drilled before 2007 to 2,864 wells drilled during the 2007–2010 period of our study, and with continued rapid expansion to as many as 80,000 forecasted [2].

Several recent reviews summarizing the evolving UGD process describe the potential for adverse health effects and delineate challenges that have contributed to as yet minimal understanding of public health impact [1, 3–4]. UGD is a dynamic process encompassing preparation of the site, well development and production, the removal of wastes and the downstream distribution of gas [1]. The well is drilled vertically into a shale layer often 1.5 km underground and then turned laterally within the shale layer for another 2–3 km before holes are blown at intervals in the pipe. This is followed by the high-pressure injection of approximately 5 million gallons of water to hydraulically fracture the shale layer, allowing the release of gas tightly bound to the shale. Added to this water is a complex mixture, including approximately 15% of a physical agent (usually silica) to prop open the fractures and about 0.5–2.0% of an evolving mixture of about 6–10 chemicals (e.g., surfactants, biocides, metal chelators, and others), that enhance release and flow of the gas. Return or flowback fluids include mixtures of the hydrofracturing agents, hydrocarbon products (methane and other volatile organic hydrocarbons including benzene) and, of particular toxicological significance, naturally occurring agents dissolved from the shale bed (e.g., brine, radionuclides, arsenic, barium, strontium and other metals) [5–6]. Over a thousand diesel truck trips are usually required for site preparation, bringing hydrofracturing fluids and disposing of the approximately 1–2 million gallons of fluid that flows back from the well. In the western US, flowback fluids are generally rapidly disposed of in deep underground injection wells. Such wells are uncommon in Pennsylvania. UGD operators first discharged to publically owned treatment works, which treated the wastewater and discharged to the regional rivers until it was determined that this practice was associated with increasing concentrations of bromine and other contaminants in drinking water pulled from the rivers [7–8]. Next, the flowback waters were transported to deep underground injection wells in Ohio. However, the resultant mild earthquakes in Ohio have led to a variety of attempted solutions to deal with these flowback fluids on the surface, including impoundments and recycling, thereby increasing the opportunity for human exposure [9]. This continues to be the current situation in Pennsylvania. As flowback fluids also contain hydrocarbon product, they can be a source of air pollution. Esswein et al. recently reported that workers involved with waste fluids could be exposed to levels of benzene above allowable occupational health levels [10]. This is pertinent as benzene in air has been associated with adverse birth outcomes [11].

Wells can be hydrofractured intermittently on multiple occasions to stimulate product flow. A more continuous process of product development occurs in region-specific patterns. This includes condensate tanks and glycol dehydrators to separate dry (methane) and wet (higher carbons such as ethane) gas components of product and diesel fuel operated compressors (to liquefy gas for shipping via pipelines) [12]. As such, concern about air pollution is both direct (flaring of methane gas at well heads, controlled burning of natural gas and release of VOCs including benzene, toluene, ethylbenzene and xylene) and indirect (traffic, diesel operated compressors).

Major challenges in assessing and quantifying environmental, ecological and human health related effects (existing and potential) of UGD exist largely due to the dynamic and complex nature of the evolving UGD process itself as well as differences in geology between site locations, UGD technique and community demography. Together, these factors make it difficult to compare experiences, historically and concomitantly, within and between regional efforts. Several recent studies have provided measurements of likely pollutants, focusing on hydrocarbons found in air [13] or on thermogenic methane found in shallow drinking water sources [12, 14–15]. A study in Colorado revealed that those living within 0.5 miles of a well were exposed to air pollutant levels, including benzene, that significantly increased non-cancer risk [16]. However, there is still a lack of information linking potential exposures with public health risks, which led the State of New York to the following declaration: “Until the science provides sufficient information to determine the level of risk to public health from HVHF and whether the risks can be adequately managed, HVHF should not proceed in New York State” [17].

The embryo/fetus is particularly sensitive to the effects of environmental agents [18]. A host of environmental and behavioral risk factors have been identified and linked to low birth weight and prematurity. They include most notably cigarette smoking [19–20], maternal occupational exposures to metals [21–22], and recently PM_2.5_ and ozone [13, 23–24]. The mechanism is thought to be one involving oxidative stress or inflammation [25]. Xu et al. have noted a relationship in southwestern Pennsylvania of low birth weight and PM_2.5_ [23]. The strength of using birth outcomes is the availability of data and the ability to capture the critical time of exposure and linkage to outcomes within the nine month period [26]. McKenzie et al. used a retrospective cohort design and exposure estimates from an inverse distance weighted (IDW) approach to explore associations between maternal residential proximity to hydraulic fracturing sites in Colorado and birth outcomes [27]. They found an increase in the prevalence of congenital heart defects and, to a lesser extent, neural tube defects with increasing exposure to natural gas extraction. They also found an increase in birth weight associated with well density.

We adapted the epidemiological and geographic information systems (GIS) approaches of McKenzie et al. [27] to explore the potential effects of UGD on infants born to mothers living in Southwestern PA where unconventional drilling of the Marcellus Shale has been rapidly expanding. The objective of the present study is to use readily available data on birth outcomes for Southwestern Pennsylvania to investigate the relationship of proximity to UGD and perinatal outcomes for 2007 to 2010.

## Methods

Natural gas well and birth data were collected for Butler, Washington and Westmoreland counties in PA for the years 2007 to 2010. The UGD locations were obtained from the Pennsylvania Department of Environmental Protection (PADEP), that defines UGD as wells having both a lateral component and hydraulic fracturing, a process relatively new to Pennsylvania until 2005 [2]. The PADEP dataset also includes information on drilling commencement dates, known as the SPUD date, and well status (active, abandoned, etc.) [2]. Birth data for these counties were obtained using information from birth certificates, which had also been geocoded by the Pennsylvania Department of Health (PADOH) Bureau of Vital Statistics. This study was approved by the University of Pittsburgh Institutional Review Board (IRB number PRO12060174). Individual data on these births was accessed through a password protected application with the PADOH. Information was abstracted regarding maternal risk factors (age, education, cigarette smoking history, use of Women, Infant and Children/WIC assistance, gestational diabetes, prenatal visits, pre-pregnancy weight, and birth parity) as well as gestational age and gender of child at birth [28]. Multiple births, records without a valid geocode (X, Y coordinate), and those with missing birth outcome and demographic information were excluded from the analysis. Exact point distances between singleton-birth residences with complete information and natural gas wells were calculated using ArcMap (version 10.1; ESRI Inc., Redlands, CA).

We calculated an inverse distance weighted (IDW) well count for each mother living within 10-miles of UGD to account for both the number of unconventional wells within this buffer as well as distance of each well from the mother’s residence [27]. This metric, shown below in Eq 1, gives greater weight to unconventional wells closest to the mother’s residence:

$$\text{IDW well count}=\sum_{i=1}^{n}\frac{1}{di}$$ (1)

where the IDW well count is the inverse distance weighted count of unconventional wells within a 10-mile radius of maternal residence in the birth year, *n* is the number of existing unconventional wells within a 10-mile radius of maternal residence in the birth year, and *d*_*i*_ is the distance of the *i* ^*th*^ individual well from the mother’s residence. For example, a mother’s residence that has two wells, both 0.5 mile away, would have an IDW well count of 4. Mothers were categorized into exposure quartiles according to their IDW well counts:

Group 1: IDW Well Count >0 but <0.87
Group 2: IDW Well Count ≥0.87 but <2.60
Group 3: IDW Well Count ≥2.60 but <6.00
Group 4: IDW Well Count ≥6.00

Three indicator variables were created, using the first quartile (Group 1) as the referent group. The 10% of births that did not live within 10 miles of UGD were eliminated from the analysis due to notable sociodemographic differences; these mothers were more African American (7% compared to 3%), smoked more during pregnancy (25% versus 20%), and had a higher proportion receiving WIC assistance (41% versus 32%).

The outcomes assessed were continuous birth weight, small for gestational age (SGA), and prematurity (gestational age <37 weeks). To identify SGA births, birth weights were normalized to gestational age and estimates of SGA were deduced from nomograms identifying elements of fetal growth (SGA <10% of predicted weight for a given gestational age and gender) [29]. Mean birth weights in each group were compared using analysis of variance (ANOVA), and proportions of SGA and premature infants were compared using chi-square tests. Outcomes were modeled using multivariate linear regression (continuous birth weight) or logistic regression (SGA and prematurity). All models were adjusted for gender of the child and mother’s age, education (8^th^ grade or less; 9^th^-12^th^ grade, no diploma; high school graduate or GED completed; some college credit, but not a degree; associate degree; bachelor’s degree; master’s degree; doctorate or professional degree), pre-pregnancy weight, prenatal care (1 if at least 1 visit; 0 otherwise), smoking (1 if smoked at all during pregnancy; 0 otherwise), gestational diabetes (1 if present; 0 otherwise), WIC (1 if received; 0 otherwise); African American (1 if yes; 0 otherwise) and parity (first child; second child; third child; fourth child or greater). The model for continuous birth weight was also adjusted for gestational age to account for the downward shift in birth weights accompanying shorter gestational ages due to earlier obstetric intervention observed in our dataset from the PADOH as well as nationally [30]. All statistical tests were performed using IBM SPSS Statistics 21 and assessed at a significance level of α = 0.05.

## Results

### Descriptive statistics

**Fig 1:**
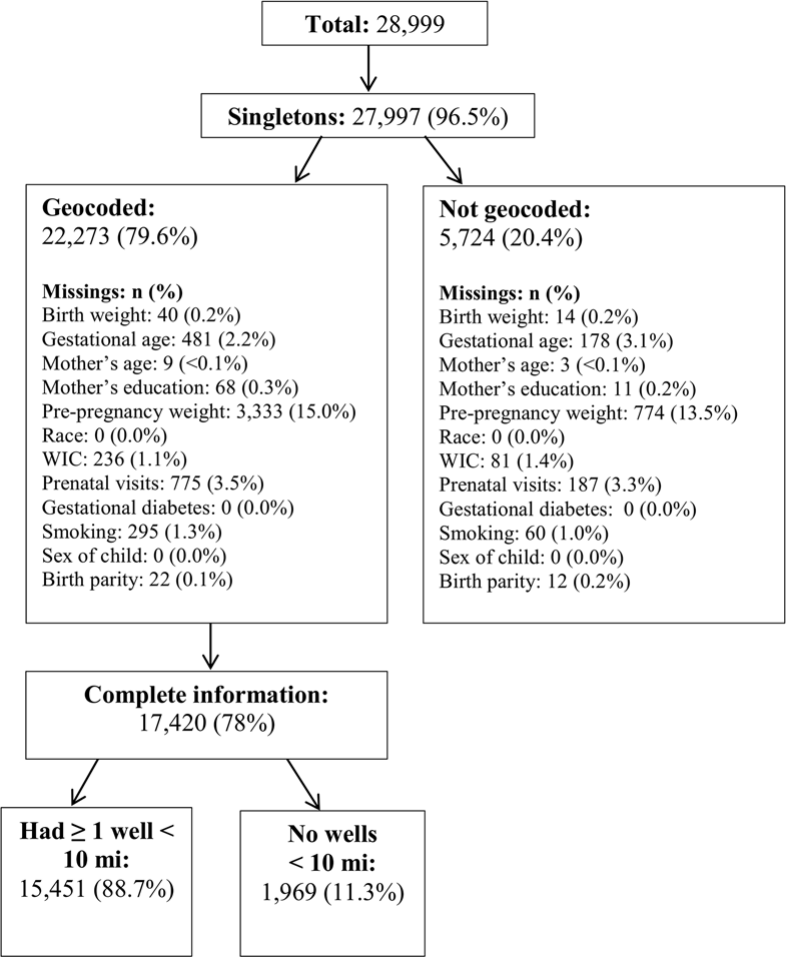
Flowchart of sample sizes and missing data for births in Butler, Washington, and Westmoreland Counties 2007–2010.

This analysis included 509 active unconventional natural gas wells in Butler, Washington and Westmoreland counties from 2007 to 2010, representing 18% of the state-wide total of 2,864 [2]. Fig 1 shows the steps used to eliminate unavailable and missing birth certificate data, leading to the final sample of births with complete information. There were 28,999 total births in these three counties from 2007 to 2010, and 27,997 (97%) of these were singleton live births. Out of the singleton birth residences, 5,724 (20%) were not geocoded to an X,Y coordinate and, since the dataset did not include an address or zip code for the mother’s residence, were excluded from the analysis. This left 22,273 singleton births available for further analysis in ArcGIS. Birth weight was missing for 0.2% of these geocoded singleton births, and gestational age was missing for 2.2%. Mother’s age, mother’s education, and birth order were missing for less than 1% of births. Pre-pregnancy weight was missing for 15% of mothers, WIC assistance for 1.1%, the number of prenatal visits for 3.5%, and information on smoking for 1.4%. The remaining 17,420 births had complete geographical and birth certificate information. Of these, 15,451 (89%) had at least one well within 10-miles of the mothers residence.

Table 1 shows the demographics of these 15,451 infant-mother pairs by quartile (the referent group (first quartile) and three exposure quartiles) as well as the proportions of SGA and premature infants in each group. Mother’s education and parity were categorized into 8 and 4 groups, respectively; results are presented for percentage that completed high school/GED and first child. There were no significant differences in prenatal care, gestational diabetes, child gender, or parity between the referent and exposure quartiles. Differences in gestational ages and mother’s ages between the four groups were small but statistically significant. Mother’s education, pre-pregnancy weight, race, WIC assistance, and smoking were also statistically different between the four groups. Chi-square analyses showed statistically significant differences in the proportions of SGA and preterm births. All proportions of SGA were significantly less than the 10% expected for the population [31] but were similar to the general population (regardless of proximity to well) in various counties in our study.

**Table 1:** Maternal and Child Risk Factors.

Factor	Total N = 15,451	Referent (First Quartile)^a^ N = 3,604	Second Quartile^a^ N = 3,905	Third Quartile^a^ N = 3,791	Fourth Quartile^a^ N = 4,151
Mother’s age (years)^b^	28.6 ± 5.8	28.8 ± 5.8	28.7 ± 5.8	28.6 ± 5.7	28.3 ± 5.8
Mother’s Education (% high school graduate/GED) ^b^	22.7%	22.1%	22.5%	22.6%	23.6%
Pre-Pregnancy Weight (lbs) ^b^	153.8 ± 39.1	152.6 ± 38.2	152.9 ± 38.2	155.2 ± 40.2	154.7 ± 39.9
Race (% African American) ^b^	3.0%	2.6%	2.0%	3.4%	4.1%
WIC (% assistance) ^b^	32.1%	29.6%	31.0%	33.6%	34.1%
Prenatal care (% at least one visit)	99.5%	99.5%	99.5%	99.5%	99.3%
Presence of gestational diabetes	4.1%	4.7%	3.7%	4.3%	3.9%
Cigarette smoking during pregnancy^b^	20.0%	19.6%	18.8%	19.9%	21.7%
Gestational age (weeks) ^b^	38.7 ± 1.9	38.6 ± 1.9	38.8 ± 1.8	38.7 ± 1.9	38.7 ± 1.9
Birth weight (g) ^b^	3345.8 ± 549.2	3343.9 ± 543.9	3370.4 ± 540.5	3345.4 ± 553.5	3323.1 ± 558.2
Small for gestational age^b^	5.5%	4.8%	5.2%	5.6%	6.5%
Premature^b^	7.7%	8.0%	6.7%	8.4%	7.9%
Congenital anomalies^b^	0.5%	0.3%	0.7%	0.4%	0.5%
Percent female	48.5%	48.7%	48.3%	48.6%	48.5%
Birth parity (first)	42.7%	42.8%	41.7%	42.2%	44.1%

^a^Referent (First quartile), <0.87 wells per mile; Second quartile, 0.87 to 2.59 wells per mile; Third quartile, 2.60 to 5.99 wells per mile; Fourth quartile, ≥6.00 wells per mile.

^b^Difference between quartiles is significant (p<0.05).

### Model Results

Table 2 shows the multivariate linear regression results for birth weight, adjusted for mother’s age, education, pre-pregnancy weight, gestational age, child gender, prenatal visits, smoking, gestational diabetes, WIC, race, and birth order. After accounting for these factors, we found that infants in the highest (fourth) exposure quartile tended to have lower birth weights than those in the referent group (p = 0.02). There were no significant differences in birth weight between the other exposure quartiles and the referent group. In accord with our current understanding [32], higher birth weights were associated with mothers that were younger, more educated, had higher pre-pregnancy weights, had more prenatal care, did not smoke during pregnancy, had gestational diabetes, did not receive WIC, were Caucasian, and had previous children. Higher birth weights were also associated with longer gestational ages and being male.

**Table 2:** Multivariate Linear Regression of Birth Weight and Proximity.

Model	Unstandardized Coefficients		Standardized Coefficients	t	Significance (P)
	*B*	*Standard Error*	*Beta*		
Constant	-3711.86	93.06	-39.88		<0.01
Mother’s Age	-2.95	0.77	-0.03	-3.82	<0.01
Mother’s Education	17.88	2.72	0.05	6.58	<0.01
Pre-Pregnancy Weight	2.01	0.09	0.15	23.37	<0.01
Gestational Age	172.64	1.97	0.56	87.51	<0.01
Female	-133.90	6.63	-0.12	-20.19	<0.01
Prenatal Care	127.07	51.53	0.02	2.47	0.01
Smoking During Pregnancy	-184.69	9.07	-0.14	-20.37	<0.01
Gestational Diabetes	33.57	16.82	0.01	2.00	0.05
WIC	-27.44	8.62	-0.02	-3.18	<0.01
Race	-146.22	19.88	-0.05	-7.36	<0.01
Birth parity	65.89	4.01	0.12	16.41	<0.01
Low^a^	10.55	9.52	0.01	1.11	0.27
Medium^a^	-0.48	9.59	0.00	-0.05	0.96
High^a^	-21.83	9.39	-0.02	-2.32	0.02

^a^Low, Second quartile to referent; Medium, Third quartile to referent; High, Fourth quartile to referent.

**Fig 2:**
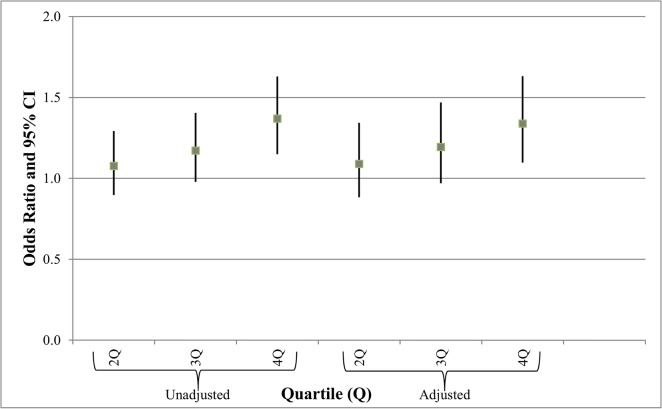
Unadjusted and adjusted odds ratios (OR) and 95% confidence intervals (CI) for small for gestational age (adjusted for mom’s age, mom’s education, pre-pregnancy weight, gender of infant, prenatal visits, smoking during pregnancy, gestational diabetes, WIC, race, and birth order). Key: Referent (First quartile), <0.87 wells per mile; Second quartile (2Q), 0.87 to 2.59 wells per mile; Third quartile (3Q), 2.60 to 5.99 wells per mile; Fourth quartile (4Q), ≥6.00 wells per mile.

Fig 2 shows the unadjusted and adjusted odds ratios (OR) and 95% confidence intervals (CI) for SGA. The steady increase in SGA across quartiles (Table 1) resulted in a progressive increase in odds ratios for SGA (unadjusted or adjusted), suggestive of a dose-response relationship. In the adjusted model, the highest exposure group compared to the referent reached significance (OR = 1.34, 95% CI = 1.10–1.63).

**Fig 3:**
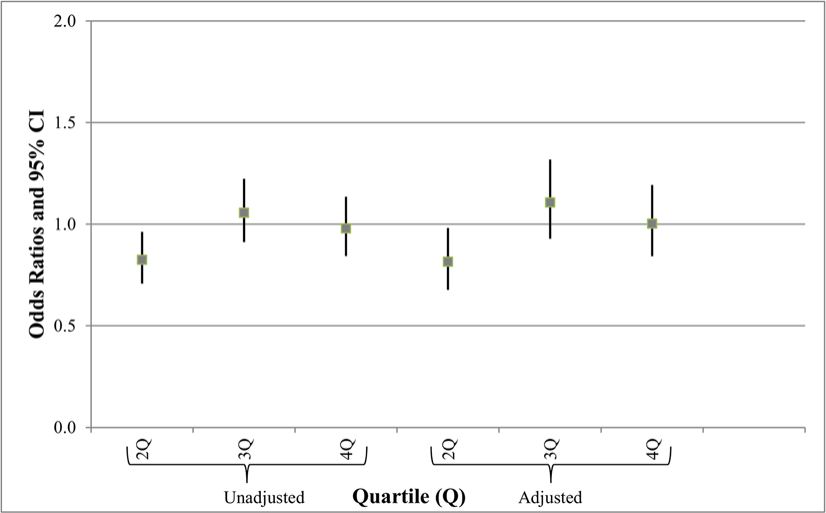
Unadjusted and adjusted odds ratios (OR) and 95% confidence intervals (CI) for prematurity (adjusted for mom’s age, mom’s education, pre-pregnancy weight, gender of infant, prenatal visits, smoking during pregnancy, gestational diabetes, WIC, race, and birth order). Key: Referent (First quartile), <0.87 wells per mile; Second quartile (2Q), 0.87 to 2.59 wells per mile; Third quartile (3Q), 2.60 to 5.99 wells per mile; Fourth quartile (4Q), ≥6.00 wells per mile.

Fig 3 shows the unadjusted and adjusted odds ratios and 95% confidence intervals for prematurity. Prematurity was associated with mothers that were older, less educated, had no prenatal care, smoked, had gestational diabetes and had no previous births. Male babies were also more likely to be premature than females. There was no significant effect of well density on prematurity except for a slightly lower proportion of premature infants born to mothers in the second exposure quartile compared to the referent (adjusted OR = 0.82, 95% CI = 0.68–0.98).

## Discussion

We accessed public records of UGD and birth and used a geographic information system that enabled proximity and density of nearby UGD to be used as a surrogate for exposure. Based on this latter estimate, we identified four groups of mothers of comparable size that gave birth in the study period (2007–2010) in three counties in Southwest Pennsylvania with high levels of UGD activities. These four groups were relatively similar in various determinants of maternal and child risks for perinatal outcomes but had different levels of exposure (i.e. IDW well count) (Table 1). The information was readily compatible for multivariate linear and logistic regression analysis in which covariates of risk could be accounted for (at least within limits of available birth certificate data in Pennsylvania) and contribution of exposure could be assessed. Even when the SGA births were removed, a small but significant decrement in mean birth weight by quartile of exposure remained (p<0.05). McKenzie et al. were able to explore subsets of congenital anomalies and neural tube defects [27], but our dataset had insufficient power to explore such birth defects.

### Comparison of existing studies on UGD and perinatal outcomes

This analysis adds to possible health impact concerns recently described by McKenzie et al. in which there was an increase in birth defects associated with proximity to UGD in rural Colorado [27]. In contrast to the McKenzie et al. study [27], our observation of a decrement in birth weight in the highest exposure group is similar to preliminary reports of two other studies, including the original thesis work of Elaine Hill [33] and a recent abstract [34]. The differences in these studies on effects of UGD on birth weight from Colorado (where proximity and density were associated with a protective effect) underscore the importance of assessing health impacts in a region-specific fashion.

Geological differences are known to account for differences in flowback water composition in different shale gas areas [35]. A notable regional difference between Colorado and Pennsylvania is that the disposal of flowback fluids is far more likely to lead to human exposure in Pennsylvania where deep underground injection has not been feasible [6]. Surface disposal sites are not readily available for geolocating, and thus could not be used in our IDW model. However, impoundments and other sites to which the flowback water is piped or trucked are likely to be near drilling sites, particularly when there are multiple sites in the area, and impoundments have been demonstrated to leak [6, 8]. Therefore, the IDW model is still likely to be representative of exposure risk. There are also important regional differences within Pennsylvania that may be pertinent to a comparison of our findings with those of other studies. Southwestern Pennsylvania is a “wet gas” area, which contains far higher levels of benzene and other relatively higher weight shale gas components than do the “dry gas” areas of the rest of the Marcellus Shale regions of the state. The management of flowback fluids presents a risk of air pollution as well as water pollution. Studies with cooperating industries have shown very wide variation from site to site in methane emissions, and in worker benzene exposures [11, 36].

McKenzie et al. [27] established criteria to restrict their analysis to rural areas, thereby minimizing the contributions of other industries, traffic, congestion and other confounding influences of a more urban environment. Although UGD in Southwestern PA does not include the most dense areas of Allegheny County, the population density in the counties we studied surrounding Pittsburgh are greater than rural Colorado [37]; thus, our assessment of exposure likely included different contributing sources of confounding pollution and other variables. McKenzie et al. [27] also included impact of altitude that is important in Colorado but can be overlooked in the comparatively modest elevations in Southwestern PA. Non-white mothers were excluded in their analysis (as it was too small a group within existing cohorts) and their referent group was individuals >10 miles from UGD [27]. This group of mothers (those >10 miles) in the present study was composed of a somewhat different demographic of women than those living within 10 miles of UGD and were therefore excluded from the analysis; most notably, these mothers were more African American (7% compared to 3%), smoked more during pregnancy (25% versus 20%), and had a higher proportion receiving WIC assistance (41% versus 32%) (see Table 3). In our study, 20% of mothers reported smoking during pregnancy (see Table 1) and, although slightly higher than the overall prevalence for the state of Pennsylvania (15%), it is similar to other reports of smoking during pregnancy for the counties and the time period under study [38]. According to the Pennsylvania Department of Health, the percent of mothers that smoked during pregnancy from 2010 to 2012 was 15% in Butler, 22% in Washington, and 20% in Westmoreland [38]. In a random sample of 5,007 birth certificates from 2005 to 2009 we obtained from the PADOH for a separate study, the proportions of mothers that smoked prior to and during pregnancy were also higher than the state: 20% for Butler, 32% for Washington, and 29% for Westmoreland.

**Table 3:** Maternal and Child Risk Factors for Geocoded versus Not Geocoded Residences and Those With versus Without at Least One Well Within 10-miles.

Factor	Geocoded N = 22,273	Not geocoded N = 5,724	<10-miles N = 15,451	≥10-miles N = 1,969
Mother’s age (years)	28.5 ± 5.8	28.1 ± 6.0	28.6 ± 5.8	27.5 ± 5.9
Mother’s Education (% high school graduate/GED)	23.3%	25.6%	22.7%	27.4%
Pre-Pregnancy Weight (lbs)	154.1 ± 39.4	153.6 ± 39.4	153.8 ± 39.1	156.5 ± 41.9
Race (% African American)	3.5%	3.4%	3.0%	7.2%
WIC (% assistance)	33.2%	36.1%	32.1%	41.3%
Prenatal care (% at least one visit)	99.4%	99.1%	99.5%	99.4%
Presence of gestational diabetes	4.2%	4.4%	4.1%	4.4%
Cigarette smoking during pregnancy	20.9%	22.1%	20.0%	25.7%
Gestational age (weeks)	38.7 ± 1.9	38.7 ± 2.0	38.7 ± 1.9	38.5 ± 2.2
Birth weight (g)	3343.0 ± 553.9	3333.6 ± 558.9	3345.8 ± 549.2	3319.8 ± 594.8
Percent female	48.5%	50.0%	48.5%	48.5%
Birth parity (first)	42.6%	43.2%	42.7%	42.0%

Like McKenzie et al. [27], we were persuaded that previous experience with multiple fixed sources of pollution and birth outcomes suggests that inverse density is the best surrogate for maternal exposure [39–40]. Further, when we repeated the analyses using IDW well count as a continuous measure, the associations between increased exposure and smaller birth weights and increased odds of SGA (OR = 1.009, 95% CI = 1.003–1.015) remained significant (p<0.01). A sensitivity analysis of 2010, the year with the most UGD activity in our study period, also showed an association between increased exposure and decreasing birth weights (p = 0.03). A reanalysis (data not shown) adding county (categorically) to the adjusted linear regression led to similar conclusions regarding: a) association of lower birth weight and increased well density for the fourth quartile (p = 0.02); and b) increased odds of SGA for the highest exposure group (OR = 1.34, 95% CI = 1.10–1.63, p = 0.004).

Two other concomitant studies have findings similar to ours concerning birth weight. The PhD thesis of Elaine Hill at Cornell University compared birth outcomes for mothers who resided in regions in Pennsylvania in proximity to wells as a function of time (before and after permit and SPUD) [33]. Their model employed a difference-in-differences approach to compare groups that lived near permitted wells versus groups near permitted wells that underwent further development. An increase in prevalence of low birth weight at gestation and reduced 5 minute APGAR scores was reported while no impact on premature birth was detected for offspring of mothers living 1.5 miles or less from gas development [33]. In an abstract presented at a recent Annual Meeting of the American Economic Association, Currie et al. noted that proximity (within 1.5 miles) to a well increased low birth weight at term as measured in a multi-state sample [34]. Our study is the only one that is specifically limited to counties with intensive shale gas activities in Southwestern PA, thereby minimizing the heterogeneity of demography, geology, climate and other confounding variables.

It is only in recent years that drilling technology has rapidly advanced to be able to obtain substantial levels of natural gas tightly bound to deep underground shale layers. This continually evolving technology greatly differs from the past in using perhaps 5 million, rather than 50,000 gallons of hydrofracturing fluid under much higher pressures for each well; in having an evolving suite of hydrofracturing chemicals, with over 500 having been used; in laterally bending the well within the shale layers for greater than a kilometer; in drilling in multiple directions from the same well head from larger drill pads for sequential periods of six months or longer; and in many other technological advances. Recent reviews of shale gas issues in the United States, Canada and Europe have been consistent in commenting on the lack of health-related information [1, 4].

### Limitations

This investigation is semi-ecological in nature. We had individual data on birth outcomes and risk factors; however, the final analysis grouped mothers into exposure categories to provide a clearer picture of possible dose-response relationships. In addition, there may be a number of unknown factors that led to our conclusion that well density was associated with lower birth weight and greater odds of SGA. As in any epidemiological study, these associations do not imply causation and are hypothesis generating only. The observed associations could be due to a contaminant related to UGD, an unknown confounding factor we were unable to account for in our analyses, or chance. Moreover, we assumed that the residence on the birth certificate was synonymous with exposure during the entire pregnancy, as we have no ability to evaluate transient occupancy of the pregnant mother. However, the counties under study have relatively stable populations. US Census data (2008–2012) for living in the same house one year and over for Butler, Washington and Westmoreland Counties shows 88.6%, 88.1% and 91.0% respectively as compared to 84.8% for the US and 87.8% for Pennsylvania [37].

Proximity is a primitive surrogate for exposure itself and is uninformative of route (water, air) or etiologic agent. Our observations were based on data deduced from the Department of Environmental Protection (DEP) of Pennsylvania and assignments of longitude and latitude only from birth certificate data. Twenty percent of the birth certificate records did not have a corresponding geocode and, since no further information on address or zip code was available, these births were excluded from the analysis. However, the sociodemographic characteristics of this group were similar to those that were geocoded (Table 3). Up until recently, pertinent information from DEP was limited to date of permit request and drilling (SPUD) and status (active, plugged or abandoned). The available well permit number provides information on production and waste data [2]. Longitude and latitude defined proximity in our analyses, and we did not probe more complex issues of geology, climate or meteorological conditions; thus, the transmigration of potential pollutants in water or air remains unclear.

Other limitations in the birth dataset included the lack of a birth month and day; we were therefore only able to identify those wells drilled during the birth year of the infant. Active drilling of a well occurs over a period of only a few months, so incorporating more specific timings of exposure will be critical in future work as further data become available as to the time period during which air or water exposures are most likely. Birth weight data are reasonably precise as derived from birth certificates, but such certificates appear less reliable for gestational age [41], so derived information such as SGA may be spuriously affected. We also relied on birth certificates to incorporate non-exposure relative risks for mother and child. Although it is encouraging that in multivariate analyses, many of these contributing factors affected outcomes in a predictable fashion [32], incomplete information on many of these factors may have affected our conclusions in Table 2 and Figs 2 and 3. For example, socioeconomic status was inferred by use of assistance via WIC; smoking was neither quantitatively assessed nor confirmed beyond self-reporting; the details of prenatal care, co-morbidities and nutritional status are not on birth certificates. As such, larger studies that include medical records will be helpful.

The relative monotonic increase in SGA (Table 1) and odds ratios for SGA (Fig 2) lends credence to the possibility that this association is indeed related to increased exposure to aspects of UGD. Similarly, a significant decrease in birth weight, after adjusting for covariates, was discernable only in the highest exposure quartile (Table 2). In contrast, changes in odds ratios for prematurity were not significant, except for a very small protective effect in the second quartile (Fig 3).

If the association of lower birth weight and proximity to well is indeed secondary to environmental exposure, then identifying the route of exposure and the agents, alone or in combination, is a critical and challenging next step. In the preliminary study of Currie et al. [34], no differences between mothers with access to public or well water was found, suggesting that exposures may not be water derived. Air pollution is well known to affect perinatal outcomes [13, 23–24, 42], and a meta-analysis of 62 studies recently pointed to particulate matter, carbon monoxide and nitrogen dioxide [43]. Potential UGD derived air pollutants that are known to be associated with low birth weight include diesel exhaust [43], heavy metals [21–22, 44], benzene [45] and other volatile organic compounds [46].

In conclusion, a small but significant association between proximity to UGD and decreased birth weight was noted after accounting for a large number of contributing factors available from birth certificate data in Southwest Pennsylvania. Although the medical and public health significance of this is unclear, it was noteworthy that there was a significant increase in incidence of SGA in the most exposed group. Along with the first published study on the association of increased incidence of birth defects and proximity and density of nearby wells in Colorado [27], there is a clear need for more complete studies including larger populations, better estimates of exposure and covariates and more refined medical records. The difference in outcomes as they relate to birth weight between our study and Colorado (but similar findings to ours in the original work of Hill [33] and preliminary results of Currie et al. [34]) underscores the importance of region-specific assessment of UGD impacts on public health. Although neither the route (water, air or soil) of exposure nor etiologic agents could be addressed, this study is among the first to report a human health effect associated with hydrofracturing. The embryo/fetus is particularly sensitive to the effects of environmental agents, which can have significant lifetime consequences [18]; therefore, further investigation appears warranted.
